# Correlation between academic self-efficacy and burnout originating from distance learning among nursing students in Indonesia during the coronavirus disease 2019 pandemic

**DOI:** 10.3352/jeehp.2021.18.9

**Published:** 2021-05-11

**Authors:** Ngatoiatu Rohmani, Rosi Andriani

**Affiliations:** Department of Nursing, Jenderal Achmad Yani University, Yogyakarta, Indonesia; Hallym University, Korea

**Keywords:** COVID-19, Distance learning, Nursing students, Psychological burnout, Self-efficacy

## Abstract

**Purpose:**

Distance learning, which became widespread in response to the coronavirus disease 2019 (COVID-19) pandemic, has been a burdensome challenge for students and lecturers. This study investigated the relationship between academic self-efficacy and burnout in first-year nursing students who participated in distance learning during the COVID-19 pandemic.

**Methods:**

The study included 69 first-year nursing students at Jenderal Achmad Yani University in Yogyakarta, Indonesia. Data were collected in September 2020 through self-efficacy and burnout questionnaires that were distributed via email and social media for 2 weeks. The responses were analyzed using the gamma test.

**Results:**

Most respondents were women (78.3%), with an average age of 19 years. Most nursing students had a moderate level of academic self-efficacy (72.5%), while only 13.0% of respondents had a low level of academic self-efficacy. However, 46.4% of students experienced severe burnout during distance learning. Cross-tabulation showed that students with moderate self-efficacy were more likely to experience severe burnout (24 respondents) (P<0.01 and r=-0.884). Exhaustion was the burnout dimension most closely associated with academic self-efficacy.

**Conclusion:**

Students perceived distance learning as burdensome and reported high levels of exhaustion, which may negatively impact their academic achievement. Interventions to improve academic self-efficacy may foster students’ confidence, potentially leading to reduced burnout levels. Nurse educators should reflect upon innovative learning strategies to create a favorable learning environment for nursing students.

## Introduction

### Background/rationale

Since March 2020, Indonesia has been affected by the coronavirus disease 2019 (COVID-19) pandemic, which resulted in 1.7 million cases as of May 6, 2021. The Indonesian government has implemented various measures to prevent the spread of COVID-19, including social distancing, physical distancing, and large-scale social restrictions. Distance learning, which is an example of these policies, has had a major impact on education in Indonesia [1].

The public response to the COVID-19 pandemic made it necessary to suddenly switch from face-to-face teaching and learning sessions to online learning. Online learning can involve either synchronous or asynchronous learning experiences using various internet-enabled devices, such as laptops and mobile phones [2]. The transition to online learning had particularly meaningful impacts on nursing education, as nursing students participate in hands-on clinical practice at hospitals or other health facilities. However, clinical training has been canceled, postponed, or transferred to online activities for students’ safety in the context of the COVID-19 pandemic [3]. Some students have reported feeling that online learning makes their academic load more difficult, with specific problems including the perception that lecturers merely hand out materials, instead of truly teaching, and the diminished interactions among students and lecturers, which limit the effectiveness of courses [4]. A study of dentistry students in Indonesia found that 55.8% of participants preferred classroom learning over distance learning [5].

Tight class schedules, assignments, and practice sessions are burdensome to students, independently of the pandemic-induced shift to online learning. A study published in 2016 showed that most nursing students experienced moderate to high burnout (57.0%) resulting from learning activities [6]. Another study from 2016 also found that 35.5% of nursing students experienced a high level of burnout due to the learning process [7]. Thus, it is necessary to pay attention to moderate burnout in nursing students to reduce the negative impacts of burnout.

Students who experience burnout feel emotional exhaustion, which manifests as feeling bored, sad, worried, irritable, and even depressed. If burnout is not handled well, it is expected to affect students’ achievement [8]. A study by Pamungkas and Indrawati [9] found that burnout was affected by self-efficacy, which is defined as an individual’s belief in his/her ability to manage a particular situation. In nursing education, distance learning emphasizes students’ self-directed learning, wherein students study independently to achieve predetermined learning goals. Thus, students with a high level of academic self-efficacy are expected to take more responsibility for their learning, as well as to show higher levels of confidence in solving and finishing academic assignments. In turn, this tendency towards higher responsibility and confidence may contribute to a lower risk of burnout.

### Objectives

The purpose of this study was to investigate the relationship between academic self-efficacy and burnout among first-year nursing students in Indonesia taking part in online learning.

## Methods

### Ethics statement

This study was approved by the Institutional Review Board of Jenderal Achmad Yani University (SKep/021/KEPK/III/2020). Informed consent was obtained from the students.

### Setting

The study was conducted at the Faculty of Health, Jenderal Achmad Yani University, Yogyakarta, Indonesia. The survey response data were collected in September 2020 for 2 weeks through online questionnaires sent to each student through email and social media (WhatsApp). The survey was conducted online due to the enactment of distance learning.

### Participants

The participants of this study were first-year nursing students in the academic year 2019/2020 at Jenderal Achmad Yani University, Yogyakarta, Indonesia. In total, 69 students were recruited and all agreed to participate in the study. The study was limited to 1 educational institution due to differences in methods and nursing curriculum structure across educational institutions.

### Data sources/measurements

Academic self-efficacy was measured using a questionnaire that consisted of 31 questions divided into domains of students’ ability to do assignments, mastery of lessons or assignments, and the stability of their confidence. The questionnaire was adopted from Winanti [10] with permission (Supplements 1, 2). The reliability of the questionnaire for this study was shown by a Cronbach α value of 0.965. The questionnaire is a Likert scale ranging from 1 (completely false) to 4 (completely true). The results were classified as mild (score <62), moderate (score between 62 and 93), or high (score ≥94). The Maslach Burnout Inventory-Student Survey (MBI-SS) was used to measure burnout; this questionnaire has been translated into Indonesian and modified by Laili [11] with permission (Supplement 3). The reliability value of the Indonesian-language version of the MBI-SS in this study was confirmed by a Cronbach α of 0.968. The MBI-SS consists of 3 subscales: items 5, 8, 9, 11, 15, 17, 20, and 23 for exhaustion; items 1, 3, 4, 6, 12, 14, 18, and 21 for cynicism; and items 2, 7, 10, 13, 16, 19, 22, and 24 for inefficacy. Each item is rated on a 7-point Likert scale ranging from 0 (never) to 6 (always). The results were categorized as mild (score <48), moderate (score between 48 and 96), and severe (score ≥97). The cutoffs were generated for the 3-way divisions into mild, moderate, and severe for both self-efficacy and burnout, which have been validated in the original literatures of the measurement tools.

### Study size

The minimum required sample size was calculated using an α error probability of 0.05, a power (1-β probability) of 0.80, and a correlation coefficient of 0.365 based on a previous study [12]. The minimum sample size was 59; thus, a sufficient number of respondents was included to evaluate the correlation between the study variables.

### Statistical methods

Descriptive statistics were used to explore general characteristics and levels of self-efficacy and burnout among nursing students. The gamma test was performed in the bivariate analysis to evaluate the relationship between academic self-efficacy and burnout among nursing students. The level of statistical significance was P<0.05.

## Results

### Participants

The participants included 54 women (78.3%) with an average age of 19 years. Forty-six respondents (66.7%) still lived with their parents or family, and 15 respondents (21.7%) had majors in non-science or health fields (Table 1).

### Academic self-efficacy

Academic self-efficacy was measured based on students’ ability to do assignments, mastery of lessons or assignments, and the stability of their confidence. Most of the respondents (72.5%) had a moderate level of self-efficacy, and 10 had a high level of self-efficacy (14.5%) (Table 2, Dataset 1).

### Burnout

The MBI-SS questionnaire was used to measure students’ burnout based on their feelings of fatigue due to learning demands, pessimism and disinterest in assignments and lessons, and incompetence as students. Fifty-six respondents (81.2%) experienced moderate to severe burnout, while only 13 students had mild burnout (18.8%) (Table 3).

### Correlation between students’ academic self-efficacy and burnout

Cross-tabulation showed that students with high academic self-efficacy tended to experience mild burnout, while students with moderate academic self-efficacy tended to experience moderate to severe burnout. Most students with low self-efficacy experienced severe burnout. A strong negative correlation (r=-0.884) was found between academic self-efficacy and burnout, meaning that students with higher academic self-efficacy experienced milder burnout (Table 4). Since the data were not normally distributed, the gamma correlation coefficient was chosen to perform the analysis based on an ordinal scale.

Gamma correlation analysis was also conducted to explore the correlations between academic self-efficacy with the subdomains of burnout. As shown in Table 5, significant results were found for all dependent variables (P≤0.001). Exhaustion was the burnout dimension most closely associated with academic self-efficacy.

## Discussion

### Key results

A significant relationship was found between academic self-efficacy and burnout among first-year nursing students during online learning. Students who had low academic self-efficacy were more likely to experience severe burnout and vice versa.

### Interpretation

Academic self-efficacy is related to an individual’s experience and maturity. The participants of this study were in their second semester, meaning that they were relatively inexperienced with nursing. This fact, in combination with online learning, may have affected their academic self-efficacy, which was moderate for most students (72.5%). Furthermore, the first-year students were 19 years old on average, and age also contributes to academic self-efficacy.

Students with low or moderate academic self-efficacy are less likely to be able to explore their abilities and make decisions for themselves. In this study, most students had negative answers for an item asking whether they were confident in facing all difficult challenges on all examinations. Thus, students considered themselves unable to solve problems or overcome difficulties related to lessons. Most respondents experienced fatigue, sensitivity, and irritability as signs of burnout, and even reported a decline in grades. In the current setting of distance learning, students require appropriate levels of self-efficacy to support an optimal learning process.

The findings of this study may imply that academic self-efficacy reduces the exhaustion caused by learning demands. As a dimension of burnout, exhaustion refers to exaggeratedly intense emotions and feelings of depletion of emotional resources. Students with low academic self-efficacy tended to experience more exhaustion, which was reflected by not being enthusiastic in their activities, feeling tired when they got up in the morning, feeling tense during lessons, and not focusing on completing each task.

### Comparison with previous studies

The findings of this study reinforce those of a previous study, which found that burnout among students was associated with perceived self-efficacy. A higher sense of academic self-efficacy was associated with lower perceived burnout [12]. Individuals with high academic self-efficacy are confident in their ability to modify the situations around them. As pointed out in previous research conducted in Indonesia, in difficult situations, someone with low academic self-efficacy is more likely to give up, while someone with high academic self-efficacy will invest more effort into overcoming challenges [13].

### Limitations

This study has potential limitations, including the fact that the study was conducted at a single institution, meaning that the results cannot be generalized. Additionally, the cross-sectional nature of the study design limits causal interpretations.

### Conclusion

Academic self-efficacy showed a strong negative association with burnout among first-year nursing students participating in online learning during the COVID-19 pandemic. Hence, education providers and managers should make serious efforts to improve students’ academic self-efficacy by involving both lecturers and educational staff to create a supportive academic environment. Innovations in learning should also be made to reduce students’ burnout levels during online learning.

## Figures and Tables

**Table 1.** Subjects’ general characteristics (N=69)

Characteristic	Value
Sex	
Female	54 (78.3)
Male	15 (21.7)
Age (yr)	19±0.85 (17–23)
Living situation	
Alone/with a landlord	23 (33.3)
Mother/father/family	46 (66.7)
School major	
Science	37 (53.6)
Social science	13 (18.8)
Health vocation	17 (24.6)
Non-health vocation	2 (2.9)

Values are presented as number (%) or mean±standard deviation (range).

**Table 2.** Academic self-efficacy of respondents (N=69)

Academic self-efficacy	Frequency (%)
Low self-efficacy	9 (13.0)
Moderate self-efficacy	50 (72.5)
High self-efficacy	10 (14.5)
Total	69 (100.0)

**Table 3.** Burnout of respondents (N=69)

Burnout	Frequency (%)
Mild	13 (18.8)
Moderate	24 (34.8)
Severe	32 (46.4)
Total	69 (100.0)

**Table 4.** Cross-tabulation and gamma correlation test for the relationship between respondents’ academic self-efficacy and burnout

Variable	Burnout				r	P-value
	Mild	Moderate	Severe	Total		
Academic self-efficacy					-0.884	≤0.001
Low	0	1 (4.2)	8 (25.0)	9 (13.0)		
Moderate	6 (46.2)	20 (83.3)	24 (75.0)	50 (72.5)		
High	7 (53.8)	3 (12.5)	0	10 (14.5)		
Total	13 (100.0)	24 (100.0)	32 (100.0)	69 (100.0)		

Values are presented as frequency (%).

**Table 5.** Correlations between academic self-efficacy and each dimension of burnout

Variable	Cynicism		Exhaustion		Inefficacy	
	r	P-value	r	P-value	r	P-value
Academic self-efficacy	-0.873	0.00	-0.898	0.00	-0.792	0.00

## Footnotes

**Data availability** Data files are available from Harvard Dataverse: https://doi.org/10.7910/DVN/N2MIUP Dataset 1. Response data from the 69 nursing students for the 31-item questionnaire of self-efficacy and 24-item Maslach Burnout Inventory-Student Survey, September 2020 in Indonesia.

## Supplementary materials

Supplementary files are available from Harvard Dataverse: https://doi.org/10.7910/DVN/N2MIUP

Supplement 1. Academic self-efficacy questionnaire (Indonesian version).

Supplement 2. Academic self-efficacy questionnaire (English version).

Supplement 3. Burnout survey tool (Indonesian version).

Supplement 4. Audio recording of the abstract.
