# Supplementary material for: Correlation between academic self-efficacy and burnout originating from distance learning among nursing students in Indonesia during the coronavirus disease 2019 pandemic
Source: J Educ Eval Health Prof. 2021 May 11;18:9. doi: 10.3352/jeehp.2021.18.9 (PMC8187029; doi:10.3352/jeehp.2021.18.9)
Supplement: Supplementary file 2 — Supplement 1. Academic self-efficacy questionnaire (Indonesian version). [file jeehp-18-09-suppl1.docx]

**KUESIONER *ACADEMIC SELF-EFFICACY***

**Petunjuk:**

Jawablah setiap pernyataan berikut dengan memberikan tanda ceklist **(√)** pada salah satu kolom yang sesuai dengan pilihan serta keadaan anda.

Keterangan pilihan jawabaan :

- SS = Sangat Setuju
- S = Setuju
- TS = Tidak Setuju
- STS = Sangat tidak setuju

| **NO** | **Pernyataan** | **SS** | **S** | **TS** | **STS** |
| --- | --- | --- | --- | --- | --- |
| 1 | Saya dapat mengerjakan tugas perkuliahan yang sulit dengan baik |  |  |  |  |
| 2 | Saya yakin dapat mengerjakan ujian tengah semester walau pun itu sulit |  |  |  |  |
| 3 | Apabila terjadi hambatan dalam berkuliah saya yakin dapat menyelesaikannya |  |  |  |  |
| 4 | Saya yakin rintangan yang ada selama berkuliah saya dapat menghadapinya |  |  |  |  |
| 5 | Apa bila terjadi permasalahan diperkuliahan, saya akan menghadapinya |  |  |  |  |
| 6 | Saya yakin permasalahan yang ada pada diri saya saat berkuliah saya mampu menyelesaikannya dengan tuntas |  |  |  |  |
| 7 | Saya dapat meyakinkan diri saya sendiri bahwa universitas yang saya pilih memang sesuai dengan saya potensi yang saya miliki |  |  |  |  |
| 8 | Saya dapat mengerjakan berbagai hal tanpa bantuan orang tua |  |  |  |  |
| 9 | Saya yakin studi yang saya pilih mempunyai masa depan yang baik untuk saya nantinya |  |  |  |  |
| 10 | Saya merasa semakin bersemangat ketika saya mendapatkan nilai yang tinggi pada saat ujian |  |  |  |  |
| 11 | Saya sering menjawab pertanyaan yang diberikan dosen dan saya merasa bangga |  |  |  |  |
| 12 | Dengan banyak berlatih soal saya mampu menguasai materi |  |  |  |  |
| 13 | saya yakin bahwa penguasaan materi saat perkuliahan saya, lebih baik dari pada teman-teman saya |  |  |  |  |
| 14 | Saya harus yakin mendapatkan nilai terbaik diantara teman-teman saya |  |  |  |  |
| 15 | Saya yakin dapat menyelesaikan soal-soal ujian tengah semester dan ujian akhir semester dengan nilai baik |  |  |  |  |
| 16 | Saya yakin dapat mendapatkan nilai terbaik dikelas |  |  |  |  |
| 17 | Saya dapat mengerjakan tugas –tugas mata kuliah dengan baik dari pada teman-teman saya |  |  |  |  |
| 18 | Saya yakin dapat menghadapi ujian mata kuliah sesulit apapun itu |  |  |  |  |
| 19 | Saya dapat mengerjakan setiap ujian mata kuliah tanpa harus melihat jawaban dari teman |  |  |  |  |
| 20 | Saya mampu mengerjakan ujian akhir semester tanpa bantuan dari orang lain |  |  |  |  |
| 21 | Saya yakin bahwa nilai ujian saya mendapat nilai terbaik diantara teman-teman dikelas |  |  |  |  |
| 22 | Saya yakin dapat menyelesaikan tugas yang diberikan oleh dosen secara tepat waktu |  |  |  |  |
| 23 | Saya dapat menyelesaikan tugas mata kuliah dengan semaksimal mungkin |  |  |  |  |
| 24 | Saya menunda-nunda mengerjakan tugas, yang saya rasa itu menyulitkan saya |  |  |  |  |
| 25 | Saya merasa pesimis, apabila saya di berikan tugas untuk meperbaiki nilai yang rendah. |  |  |  |  |
| 26 | Meskipun saya belajar dengan giat hasil ujian yang saya dapat tetap buruk |  |  |  |  |
| 27 | Perasaan saya biasa saja meskipun saya mendapat nilai C dalam mata kuliah yang saya ambil |  |  |  |  |
| 28 | Saya sering mencontek saat ujian karena soal yang diberikan sulit |  |  |  |  |
| 29 | Saya sering terlambat mengumpulkan tugas yang diberikan dosen |  |  |  |  |
| 30 | Saya malas untuk berkuliah karena nilai yang saya dapat selalu tidak memuaskan |  |  |  |  |
| 31 | Saya merasa malas untuk berkuliah karena matakuliah saat itu sangat tidak saya sukai |  |  |  |  |
