# Supplementary material for: Correlation between academic self-efficacy and burnout originating from distance learning among nursing students in Indonesia during the coronavirus disease 2019 pandemic
Source: J Educ Eval Health Prof. 2021 May 11;18:9. doi: 10.3352/jeehp.2021.18.9 (PMC8187029; doi:10.3352/jeehp.2021.18.9)
Supplement: Supplementary file 3 — Supplement 2. Academic self-efficacy questionnaire (English version). [file jeehp-18-09-suppl2.docx]

***ACADEMIC SELF-EFFICACY* QUESTIONNAIRE**

**Directions:**

Some statements concerning your beliefs about learning are given below. Four responses are given to each statement: 1. Exactly True 2. True 3. Not True 4. Exactly False. Carefully read each statement and decide to what extent it is true in your case. Then mark **√** in the column of the given response sheet.

Notes:

- ET = Exactly True
- T = True
- NT = Not True
- EF = Exactly False

| **NO** | **Statement** | **ET** | **T** | **NT** | **EF** |
| --- | --- | --- | --- | --- | --- |
| 1 | I can do difficult assignments well. |  |  |  |  |
| 2 | I am certain I can do mid-semester examination despite its difficulty. |  |  |  |  |
| 3 | I am confident I can solve problems during my study |  |  |  |  |
| 4 | I am confident I can face obstacles during my study |  |  |  |  |
| 5 | If any problems appear during my study, I will face them |  |  |  |  |
| 6 | I am certain I can solve any problem during my study completely |  |  |  |  |
| 7 | I can convince myself that I choose a university which meets my potentials |  |  |  |  |
| 8 | I can do various things independently (without my parents’ help) |  |  |  |  |
| 9 | I am sure I choose the study that has a good future for me |  |  |  |  |
| 10 | I feel more excited when I get high score during my examination |  |  |  |  |
| 11 | I often answer questions asked by lecturers and I feel proud with my answers |  |  |  |  |
| 12 | With more practice, I am able to gain mastery of subject materials |  |  |  |  |
| 13 | I am confident that I am better than my college classmates in terms of lesson mastery |  |  |  |  |
| 14 | I must be confident that I will get the best score among my college classmates |  |  |  |  |
| 15 | I am confident that I can finish mid-semester examination and end of semester examination with good score |  |  |  |  |
| 16 | I am confident that I can the best score in the class |  |  |  |  |
| 17 | I can do subject assignments better than my college classmates |  |  |  |  |
| 18 | I am confident that I can do any examination, however difficult it is |  |  |  |  |
| 19 | I can do any examination without cheating from other college classmates |  |  |  |  |
| 20 | I am able to do end of semester examination independently |  |  |  |  |
| 21 | I am confident that I get the best score in my examination among my college classmates |  |  |  |  |
| 22 | I am confident that I can finish tasks given by the lecturers on time |  |  |  |  |
| 23 | I can finish subject assignments as best as I can |  |  |  |  |
| 24 | I postpone doing difficult assignments |  |  |  |  |
| 25 | I feel pessimistic if I am given remedial assignments |  |  |  |  |
| 26 | My score is low despite my active learning |  |  |  |  |
| 27 | I feel normal even though I get C in the subjects taken |  |  |  |  |
| 28 | I often cheat my college classmates during the examinations because they are difficult |  |  |  |  |
| 29 | I am often late in submitting assignments given by the lecturer |  |  |  |  |
| 30 | I am lazy to attend classes because I always get unsatisfactory scores |  |  |  |  |
| 31 | I feel lazy to attend the class because I do not like the subject |  |  |  |  |
