# Supplementary material for: Correlation between academic self-efficacy and burnout originating from distance learning among nursing students in Indonesia during the coronavirus disease 2019 pandemic
Source: J Educ Eval Health Prof. 2021 May 11;18:9. doi: 10.3352/jeehp.2021.18.9 (PMC8187029; doi:10.3352/jeehp.2021.18.9)
Supplement: Supplementary file 4 — Supplement 3. Burnout survey tool (Indonesian version). [file jeehp-18-09-suppl3.docx]

**KUESIONER *BURNOUT***

PETUNJUK PENGISIAN KUESIONER

Berikut ini terdapat sejumlah pernyataan yang menggambarkan keadaan diri mahasiswa jurusan keperawatan. Anda diminta untuk memikirkan apakah keadaan tersebut pernah anda alami atau rasakan. Kemudian beri tanda silang pada salah satu alternative jawaban yang tersedia. Terdapat enam alternatif jawaban yang berkisar 0 sampai 6 yang menunjukkan seberapa sering anda mengalami keadaan tersebut. Arti dari masing-masing alternative jawaban sebagai berikut :

| Angka 0 | | : T**idak pernah a**nda rasakan | | | | | | |  |  |
| --- | --- | --- | --- | --- | --- | --- | --- | --- | --- | --- |
| Angka 1 | | : **jarang sekali**, paling tidak anda merasakan **satu kali dalam setahun** | | | | | | |  |  |
| Angka 2 | | : J**arang**, paling tidak anda merasakan **satu kali dalam enam bulan** | | | | | | |  |  |
| Angka 3 | | : **Kadang-kadang**, paling tidak anda merasakan **satu kali dalam sebulan** | | | | | | |  |  |
| Angka 4 | | : **Sering**, paling tidak anda merasakan **satu kali dalam seminggu**. | | | | | | |  |  |
| Angka 5 | | : **Sering sekali**, paling tidak anda merasakan **beberapa kali dalam seminggu** | | | | | | |  |  |
| Angka 6 | | : **Selalu**, paling tidak anda merasakan **setiap hari** | | | | | | |  |  |
| **NO** | **Pernyataan** | | **Pilihan Jawaban** | | | | | | | |
|  |  |  | 0 | 1 | 2 | 3 | 4 | 5 | | 6 |
|  | ***Exhaution*** | |  |  |  |  |  |  | |  |
| 1 | Saya merasa lelah ketika saya bangun pagi dan saya harus menghadapi hari-hari berikutnya di kampus | |  |  |  |  |  |  | |  |
| 2 | Belajaratau emngikuti pelajaran di kelas benar-benar sebuah ketegangan bagi saya | |  |  |  |  |  |  | |  |
| 3 | Saya merasa jenuh dengan pelajaran-pelajaran saya | |  |  |  |  |  |  | |  |
| 4 | Saat mengerjakan tugas kuliah saya merasa penat | |  |  |  |  |  |  | |  |
| 5 | Saya merasa bersemangat dalam mengerjakan setiap tugas | |  |  |  |  |  |  | |  |
| 6 | Saya merasa setiap tugas yang diberikan dosen merupakan tantangan yang menyenangkan untuk saya hadapi | |  |  |  |  |  |  | |  |
| 7 | Saya merasa memiliki energi yang penuh untuk menghadapi perkuliahan | |  |  |  |  |  |  | |  |
| 8 | Saya merasa focus dalam mengerjakan tugas | |  |  |  |  |  |  | |  |
|  | **Sinisme** | |  |  |  |  |  |  | |  |
| 9 | Saya merasa menjadi kurang tertarik pada jurusan saya dan saya alami sejak mendaftarkan diri di kampus | |  |  |  |  |  |  | |  |
| 10 | Saya merasa menjadi kurang antusias terhadap pelajaran-pelajaran saya | |  |  |  |  |  |  | |  |
| 11 | Saya meragukan pentingnya saya kuliah | |  |  |  |  |  |  | |  |
| 12 | Seringkali saya merasa ingin bolos kuliah | |  |  |  |  |  |  | |  |
| 13 | Bagi saya mengerjakan tugas kuliah adalah hal yang bermakna | |  |  |  |  |  |  | |  |
| 14 | Saya merasa bangga kuliah di jurusan saya saat ini | |  |  |  |  |  |  | |  |
| 15 | Saya menemukan hikmah atau pelajaran pada setiap tugas yang diberikan | |  |  |  |  |  |  | |  |
| 16 | Tugas dari dosen pada kegiatan perkuliahan memunculkan minat dalam diri saya | |  |  |  |  |  |  | |  |
|  | **Penurunan Pencapaian Prestasi Akademik** | |  |  |  |  |  |  | |  |
| 17 | Saya merasa kurang percaya diri dalam mengerjakan tugas-tugas akademik | |  |  |  |  |  |  | |  |
| 18 | Pencapaian diri dalam kuliah yang saya miliki berkurang dari sebelum-sebelumnya | |  |  |  |  |  |  | |  |
| 19 | Saya merasa gagal dalam memenuhi pencapaian diri saat kuliah | |  |  |  |  |  |  | |  |
| 20 | Menurut saya kompetensi yang saya miliki dalam perkuliahan menurun | |  |  |  |  |  |  | |  |
| 21 | Saya percaya bahwa saya memberikan kontribusi yang efektif pada kelas-kelas yang saya hadiri | |  |  |  |  |  |  | |  |
| 22 | Menurut saya, saya seorang pelajar yang baik | |  |  |  |  |  |  | |  |
| 23 | Saya telah belajar banyak hal yang menarik selama saya kuliah | |  |  |  |  |  |  | |  |
| 24 | Selama pelajaran saya merasa percaya diri bahwa saya menyelesaikan semuanya dengan efektif | |  |  |  |  |  |  | |  |
